# Experimental Characterization and Three Network Model-Based Finite Element Analysis of TPU-95A Lattice-Structured Airless Tires

**DOI:** 10.3390/polym18212752

**Published:** 2026-11-05

**Authors:** Kenan Tüfekci

**Affiliations:** Department of Mechanical Engineering, Bursa Uludag University, Bursa 16059, Türkiye; kenantufekci@uludag.edu.tr

**Keywords:** thermoplastic polyurethane (TPU), auxetic lattices, three network model, finite element analysis, airless tires

## Abstract

Thermoplastic polyurethane (TPU) filaments are increasingly used in additively manufactured structures that require large deformation and flexible mechanical response. In this study, the mechanical behavior of TPU-95A filament was experimentally characterized and implemented in finite element analyses of lattice-structured airless tire prototypes. Tensile tests were conducted at three different loading rates using a custom-made desktop testing device, and the resulting stress–strain curves were used to calibrate the PolyUMod Three Network Model (TNM) in Ansys MCalibration version 25.1. Four airless tire prototypes were then designed, comprising a conventional hexagonal lattice and three lattice configurations based on auxetic unit-cell geometries: reentrant, arrowhead, and star. The prototypes were fabricated using fused deposition modeling and subjected to compression tests to characterize their force–displacement responses. Comparison with compression experiments at a single loading rate showed geometry-dependent agreement for the four investigated lattice configurations, supporting the applicability of the filament-derived TNM parameters under the tested conditions. Numerical predictions were validated against experimental force–displacement curves using the normalized root mean square error (NRMSE). The NRMSE values were 8.23%, 11.89%, 17.61%, and 3.94% for the hexagonal, reentrant, arrowhead, and star structures, respectively. Overall, the results demonstrate the capability of the calibrated TNM-based finite element approach to predict the compression behavior of additively manufactured TPU-95A lattice structures.

## 1. Introduction

Additive manufacturing (AM) has emerged as an important alternative to conventional manufacturing because it enables the fabrication of geometrically complex, application-specific components with reduced material waste and without dedicated molds or tooling. By constructing three-dimensional parts layer by layer directly from digital models, AM facilitates economically viable low-volume production and the development of lightweight structures that may contribute to energy savings during service [1]. Its flexibility has also enabled the production of functional engineering components, including additively manufactured gripping jaws that have been successfully used in tensile-testing systems under cyclic loading conditions [2].

Thermoplastic polyurethane (TPU) has become an attractive material for additive manufacturing because it combines the processability of thermoplastics with rubber-like flexibility and the ability to undergo large deformations [3]. These properties have promoted its use in aerospace, automotive, biomedical, impact-protection, and energy-absorption applications [4]. However, TPU exhibits a strongly nonlinear mechanical response that depends not only on the magnitude of deformation but also on the strain rate. Increasing the tensile test speed of FDM-manufactured TPU significantly increased the stress response, while elongation at break was comparatively less sensitive [5]. Accurate characterization across different loading rates is therefore essential for reliable finite-element predictions of polymer components under realistic service conditions [6,7].

Recent studies have increasingly combined additive manufacturing with experimental and numerical approaches to investigate airless tires (or non-pneumatic tires, NPTs). Wang et al. [8] fabricated TPU-95A NPTs using FDM and reported that the experimental stiffness of the printed tires was lower than numerical predictions, emphasizing the influence of manufacturing effects. Andriya et al. [9] examined TPU-based auxetic NPT concepts with three cellular architectures and identified the reentrant hexagonal configuration as a promising design. Quattrocchi et al. [10] combined experimental measurements with finite element modeling of 3D-printed airless tire prototypes and showed that material properties, lattice geometry, and load-transfer conditions strongly affect structural response. Pak et al. [11] demonstrated that finite-strain models could successfully reproduce the large-deformation radial behavior of 3D-printed hexagonal airless tires. Similarly, Wyatt et al. [12] showed through compression tests on 3D-printed TPU95 honeycomb NPT segments that both lattice geometry and finite element formulation influence numerical accuracy. More recently, Zhang et al. [13] investigated 3D-printed TPU NPTs incorporating negative-stiffness and negative-Poisson’s-ratio honeycomb supports and reported deviations below 10% between experimental and numerical results. Overall, these studies demonstrate the potential of additive manufacturing for complex NPT designs while highlighting the importance of experimentally validated numerical models.

A practical limitation in such characterization is the relatively low operating speed of conventional universal testing machines. Commercial electromechanical systems commonly provide maximum crosshead speeds of approximately 500–750 mm/min, while specialized low-force desktop systems may reach approximately 3000 mm/min. Substantially higher loading rates generally require more sophisticated and costly testing equipment [14,15]. Although modified test systems have been used to investigate polymers over ranges such as 10–300 mm/min [5], a broad loading-rate capability remains particularly desirable for low-strength elastomers such as TPU, where high load capacity is less important than testing speed.

The numerical representation of TPU presents an additional challenge. Conventional hyperelastic models, including Mooney–Rivlin, Ogden, and Neo-Hookean formulations, can describe large nonlinear deformations but are intrinsically rate-independent [16]. Raj et al. [17], for example, showed that a five-parameter Mooney–Rivlin model could satisfactorily represent TPU-based composites under selected conditions, but also emphasized that calibration at a single strain rate cannot reproduce responses at substantially different rates. Real elastomers additionally exhibit viscoelastic and viscoplastic behavior, including rate-dependent stiffness, hysteresis, delayed recovery, and large-strain hardening. Consequently, more advanced constitutive formulations are required when predictions must remain valid over a wide range of deformation rates. Similar conclusions were reported by Lang et al. [18], who demonstrated limitations of conventional hyperelastic models for TPU-60A under different loading conditions.

Among advanced constitutive approaches, the Three Network Model (TNM) provides a suitable framework for representing the nonlinear and rate-dependent response of polymers. In the formulation presented by Bergström [19], the macroscopic stress is obtained from three parallel networks. Networks A and B represent two viscoplastic mechanisms with different characteristic relaxation behaviors, whereas Network C represents the equilibrium hyperelastic response. This architecture enables the model to reproduce strain-rate dependence, hysteresis, cyclic softening, and large-strain hardening within a unified formulation. The TNM has been implemented in commercial finite element environments through the PolyUMod material-model library and can be calibrated using uniaxial test data obtained at multiple strain rates. Larin et al. [4] similarly demonstrated that rate-dependent constitutive formulations can provide more accurate predictions for TPU-based structures than conventional hyperelastic approaches.

Despite extensive studies on TPU characterization, constitutive modeling, and additively manufactured lattice structures, relatively few investigations combine multi-rate material characterization, advanced constitutive calibration, and structural-level experimental validation within a single framework. This issue is particularly relevant for lattice structures, where large deformation, bending, contact, and heterogeneous strain fields occur simultaneously, and for auxetic architectures whose deformation mechanisms differ markedly from conventional cellular structures. Accordingly, the present study investigates TPU-95A through tensile tests performed at three substantially different loading rates using a purpose-developed low-load, high-speed desktop testing system. The resulting datasets are simultaneously used to calibrate the PolyUMod Three Network Model. The calibrated model is subsequently applied to four additively manufactured airless tire geometries, comprising a conventional hexagonal lattice and three lattice configurations based on auxetic unit-cell geometries: reentrant, arrowhead, and star. The experimentally measured compression responses of these structures are finally compared with finite element predictions to evaluate the transferability of the calibrated TNM from simple tensile specimens to complex lattice structures.

## 2. Materials and Methods

### 2.1. Materials

TPU-95A (Bambu) filament(Bambu Lab, Shenzhen, China) was used for tensile testing at different strain rates, calibration of a constitutive material model, and the fabrication of lattice-structured airless tires. The filament was supplied with a nominal diameter of 1.75 mm. For the tensile tests, sections obtained from the same filament spool were used directly as test specimens, whereas for the lattice structures, the standard filament form with a diameter suitable for fused deposition modeling (FDM) extrusion was used. The block copolymer structure of TPU-95A, consisting of hard and soft segments formed by the reaction of isocyanate and polyol, imparts a characteristic mechanical response that combines the processing ease of thermoplastics with rubber-like flexibility [3,4].

The airless tires were fabricated using a Bambu Lab X1 Carbon Combo 3D printer (Bambu Lab, Shenzhen, China). The printing parameters were selected based on the ranges recommended in the literature for TPU-95A and are summarized in Table 1. The selected values are consistent with the typical practices reported for TPU-95A printing by Vidakis et al. [5]. The parameters were selected by considering the need to ensure reliable feeding of the flexible filament through the nozzle without buckling, maintain adequate interlayer bonding, and preserve the geometric integrity of the lattice cells. The tire prototypes were printed with their circular cross-sections lying in the XY plane, while their 24 mm width was built along the Z direction.

**Figure 1:**
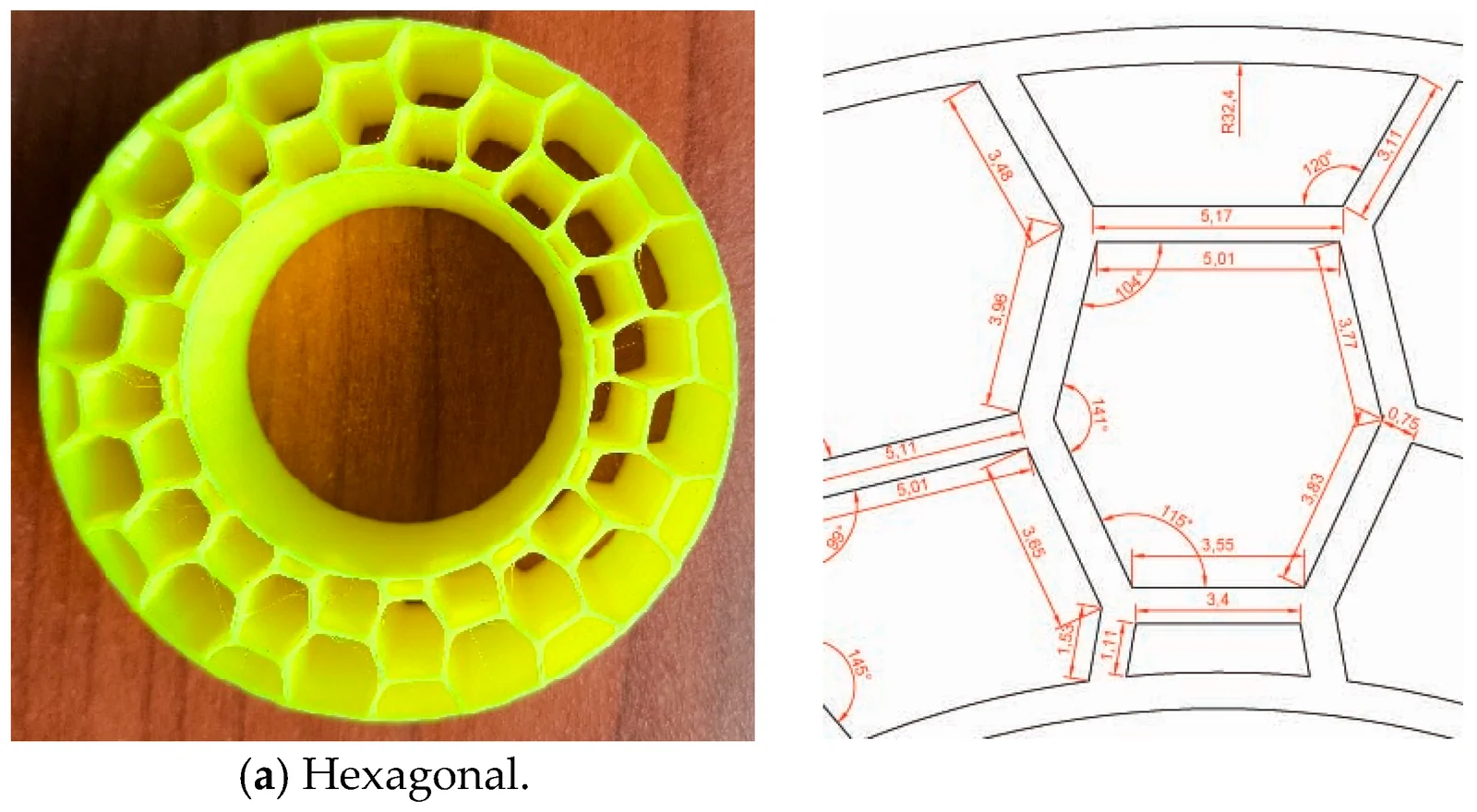

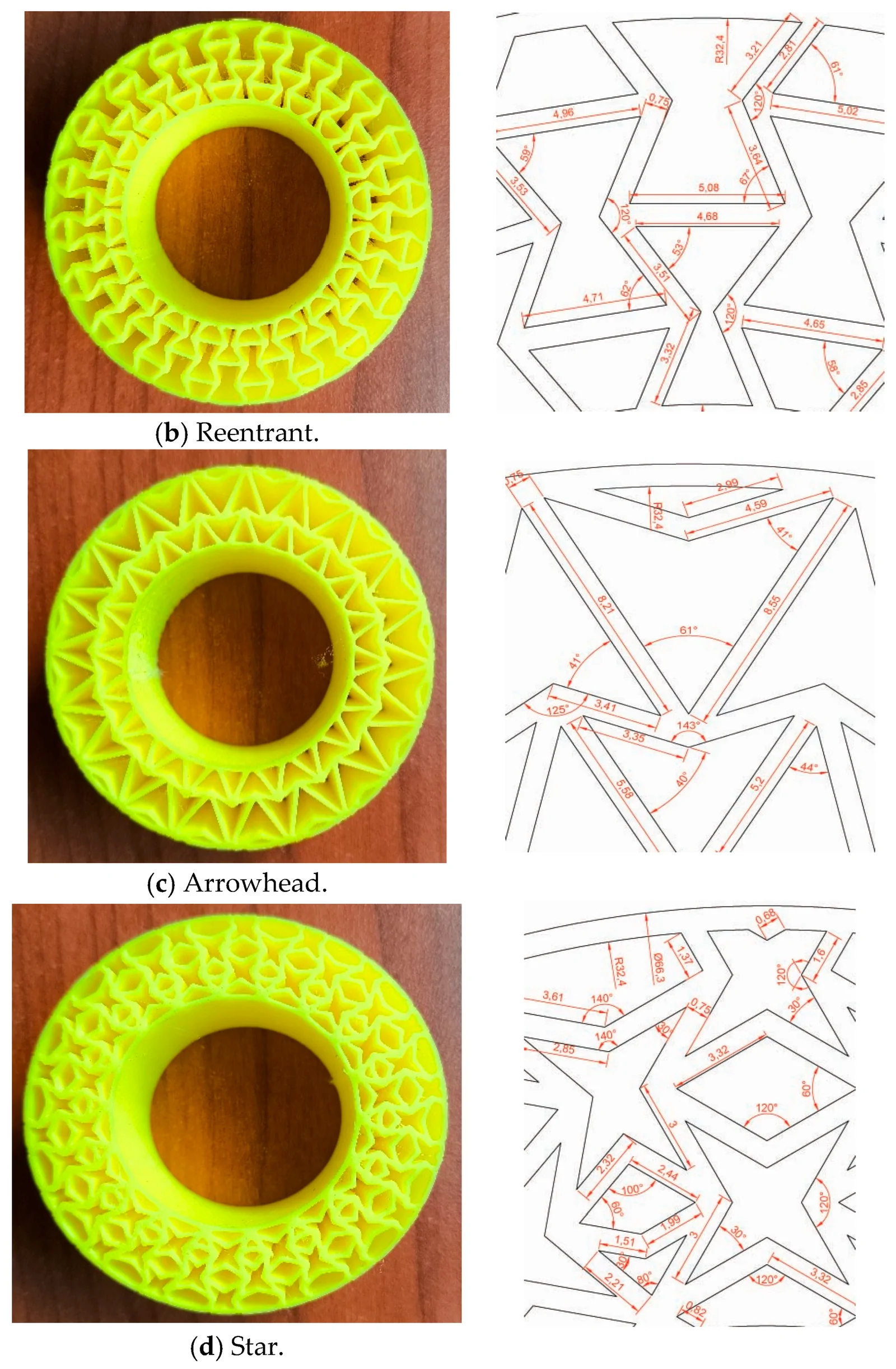
3D-printed airless tire prototypes and corresponding unit-cell geometries, showing the main dimensions of the (**a**) hexagonal, (**b**) reentrant, (**c**) arrowhead, and (**d**) star lattice designs. All dimensions are in mm.

The airless tire prototype models investigated in this study were dimensioned at an approximately 1:10 scale based on the 235/60 R15 reference tire size, which is commonly adopted as a representative dimension for passenger-vehicle applications in the automotive literature. Accordingly, the airless tire prototypes had an outer diameter of 66 mm, an inner diameter of 38 mm, and a width of 24 mm. Four airless wheel prototypes were designed: one with a conventional hexagonal lattice and three with lattice configurations based on auxetic unit-cell geometries: reentrant, arrowhead, and star. Figure 1 shows the 3D-printed airless tire prototypes, along with the main unit-cell dimensions of the four lattice designs. The lattice geometries employed in the present study were also adopted as reference models by Andriya et al. [9] in their numerical investigation of FDM-printed NPTs incorporating auxetic lattice structures.

### 2.2. Testing Systems

**Figure 2:**
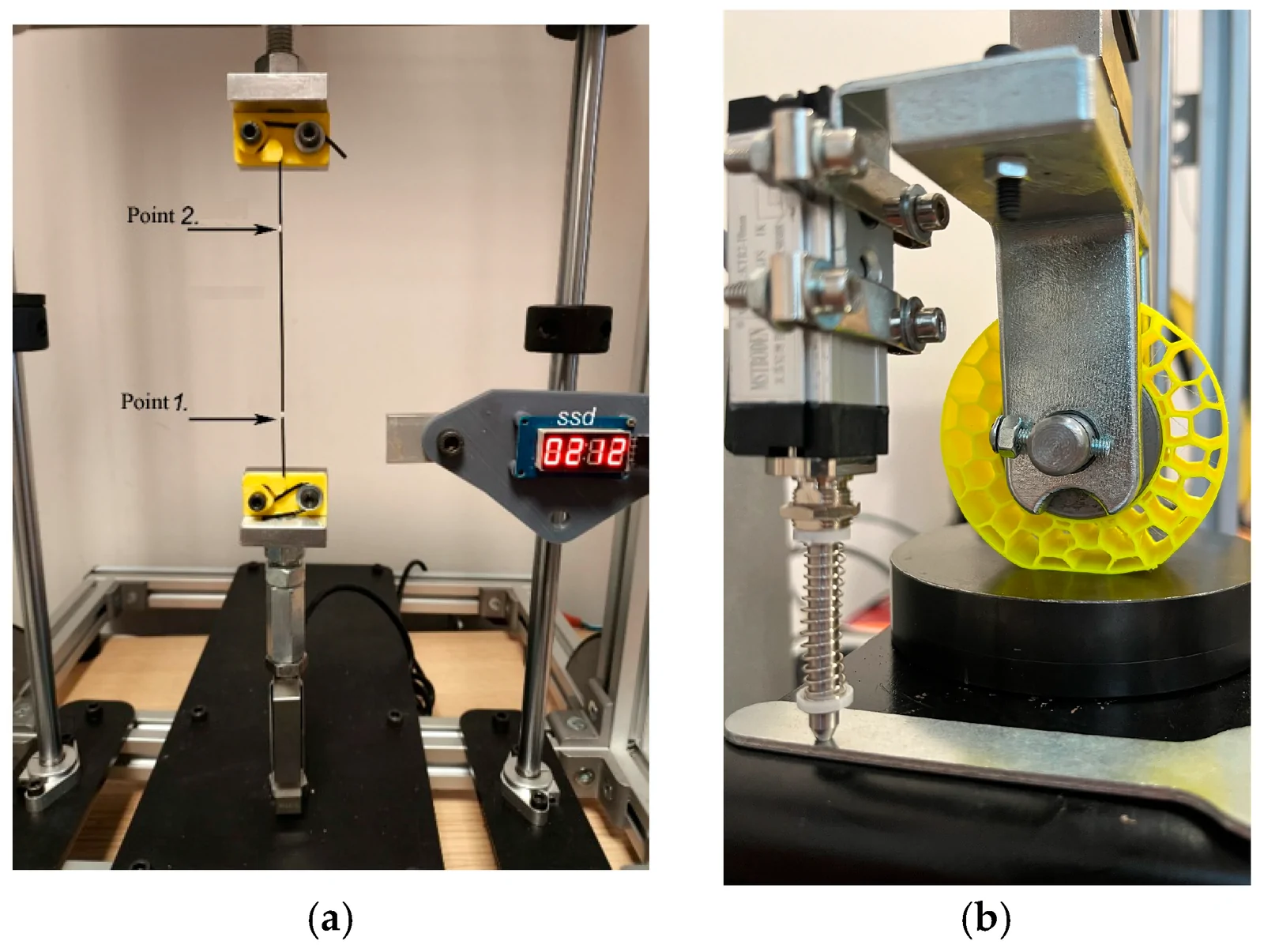
Experimental testing systems: (**a**) tensile testing system used for the mechanical characterization of TPU-95A filaments and (**b**) compression testing system used for the mechanical evaluation of 3D-printed airless tire prototypes.

Two benchtop experimental testing systems were used in this study. The first system is capable of performing uniaxial tensile tests at different crosshead speeds, with a maximum tensile speed of 6000 mm/min. A photograph of the tensile testing system is presented in Figure 2a. The second system was used to perform compression tests on the 3D-printed airless tire prototypes, and the corresponding compression testing apparatus is shown in Figure 2b. Both experimental testing systems were calibrated by Kal-Met Calibration Company (Bursa, Türkiye) to ensure the accuracy and reliability of the measurements [20,21,22].

Real-time motion control and data acquisition for both testing systems are implemented using an STM32-F407 microcontroller board (STMicroelectronics, Geneva, Switzerland) based on the ARM Cortex-M4 core. The embedded system firmware was developed in C++ using the STM32CubeIDE version 1.18.1 (STMicroelectronics, Geneva, Switzerland) environment to enable high-speed signal processing and reliable management of hardware interrupts. A graphical user interface was developed in C# using the Visual Studio environment to facilitate user–machine interaction, configure the test parameters, including crosshead speed, data acquisition rate, and target displacement, and visualize the acquired sensor data, such as force–extension responses, in real time. Continuous data communication between the microcontroller and the computer is maintained through a serial communication protocol at a rate of 100 data records per second.

In the filament tensile testing system, a 10 kg-capacity load cell was integrated to measure tensile forces with high resolution and accuracy, as the system was intended for the characterization of polymeric materials with relatively low tensile strength. The thin cross-sectional geometry and large deformation characteristics of TPU-95A filaments technically limit the use of conventional contact-type extensometers. To overcome this limitation, the strain of the specimens was determined using an image-processing-based digital point-tracking method. Before testing, two reference points were marked on the TPU-95A filament surface using acrylic paint. The initial gauge length, defined as the distance between the two marked reference points used for optical strain measurement, was approximately 50 mm. The motion of the reference points during tensile loading was recorded using a high-resolution video camera at 4K resolution and a frame rate of 120 frames per second (fps).

The recorded images were subsequently analyzed using an image-processing algorithm developed in MATLAB R2025b, and the displacement of the reference points was determined. Engineering strain values were then calculated from the measured displacement data. Meanwhile, the measured force data were simultaneously transferred to a seven-segment display (ssd) panel and incorporated into the same video frame. This approach enabled the force and displacement data to be synchronized and recorded within the same temporal reference frame (Figure 2a).

For the compression tests of the airless tire prototypes, the testing system was equipped with a 100 kg-capacity load cell for force measurement and a potentiometric linear displacement sensor for displacement measurement (Figure 2b). Compression tests on the airless tire prototypes were conducted at a crosshead speed of 10 mm/min. For each lattice configuration, three compression tests were performed on the same tire specimen at angular positions of 0°, 120°, and 240°, rotating the specimen by 120° between successive tests. The reported experimental force–displacement curve represents the mean of these three tests. These measurements capture repeated loading at different angular positions of one specimen rather than variability between independently manufactured specimens.

### 2.3. Three Network Model

The TNM consists of three molecular networks arranged in parallel, namely Network A, Network B, and Network C [23]. The total Cauchy stress tensor of the system, **T**, is expressed as the sum of the stresses contributed by the three networks:

(1) **T** = **Tᴬ** + **Tᴮ** + **T^C^**

These structural components represent different aspects of the mechanical response of the polymer.

Network A: Network A consists of elastic and viscoplastic components and contributes to both the elastic response and the strain-rate-dependent viscoplastic behavior of the material. The corresponding stress response can be calculated using Equation (2).

Network B: Network B is the second network contributing to the elastic and viscoplastic response of the material. Its effective shear modulus evolves with accumulated viscoplastic strain, thereby contributing to the representation of distributed yielding behavior. The corresponding stress response can be calculated using Equation (3).

Network C: Network C is a hyperelastic network that does not undergo viscoplastic flow and contributes to the overall elastic response of the material. The stress response of Network C can be calculated using Equation (4).

The equations given in Equations (1)–(6) represent the basic formulation of the Three Network Model and can be modified and extended for specific applications. The model parameters are defined in Table 2. The Cauchy stress is formulated using a temperature-dependent version of the eight-chain model [19].

(2) $$T_{A}=\frac{\mu_{A}}{J_{A}^{e}\lambda_{A}^{e*}}\left[1+\frac{\theta -\theta_{0}}{\hat{\theta }}\right]\frac{L^{-1}\left(\lambda_{A}^{e*}/\lambda_{\mathrm{lock}}\right)}{L^{-1}\left(1/\lambda_{\mathrm{lock}}\right)}\mathrm{dev}\left[b_{A}^{e*}\right]+\kappa \left(J_{A}^{e}-1\right)I$$

(3) $$T_{B}=\frac{\mu_{B}}{J_{B}^{e}\lambda_{B}^{e*}}\left[1+\frac{\theta -\theta_{0}}{\hat{\theta }}\right]\frac{L^{-1}\left(\lambda_{B}^{e*}/\lambda_{\mathrm{lock}}\right)}{L^{-1}\left(1/\lambda_{\mathrm{lock}}\right)}\mathrm{dev}\left[b_{B}^{e*}\right]+\kappa \left(J_{B}^{e}-1\right)I$$

(4) $$T_{C}=\frac{1}{1+q}\left\{\frac{\mu_{C}}{J\lambda^{*}}\left[1+\frac{\theta -\theta_{0}}{\hat{\theta }}\right]\frac{L^{-1}\left(\lambda^{*}/\lambda_{\mathrm{lock}}\right)}{L^{-1}\left(1/\lambda_{\mathrm{lock}}\right)}\mathrm{dev}\left[b^{*}\right]+\kappa \left(J-1\right)I\;+q\frac{\mu_{C}}{J}\left[I_{1}^{*}b^{*}-\frac{2I_{2}^{*}}{3}I-{\left(b^{*}\right)}^{2}\right]\right\}$$

(5) $${\dot{\mu }}_{B}=-{\beta (\mu }_{B}-\mu_{\mathrm{Bf}}){\dot{\gamma }}_{A},\;\;\;\mu_{B}(0)=\mu_{\mathrm{Bi}}$$

(6) $${\dot{\gamma }}_{x}={\dot{\gamma }}_{0}{\left(\frac{\tau_{x}}{{\hat{\tau }}_{x}+\mathrm{aR}(P_{x})}\right)}^{m_{x}}{\left(\frac{\theta }{\theta_{0}}\right)}^{n},\;\;\;x=A,B$$

During the calibration of the experimental data using the PolyUMod TNM in MCalibration, the arithmetic mean of the three experimental curves obtained at each strain rate was calculated and defined as the input data for the calibration process. The temperature-dependent parameters included in the TNM, but not experimentally characterized due to the isothermal conditions of the present study, were excluded from the optimization process and kept fixed at their default values. Since the experiments were conducted at a constant room temperature of 293 K, the coefficient of thermal expansion (α), the *n* parameter representing the temperature dependence of viscoplastic flow, and the temperature-dependent stiffness parameter were assigned values of zero and excluded from the calibration. Similarly, the auxiliary relative contribution parameter (*q*) obtained from biaxial tests for Network C was assigned a value of zero and excluded from the calibration. The reference temperature parameter (θ_0_) was set to 293 K and kept constant throughout the calibration process. Furthermore, since only uniaxial tensile data were used in the present study, the parameter *a*, which controls the sensitivity of viscoplastic flow to hydrostatic pressure in the TNM, was set to zero and excluded from the optimization process.

The temperature-dependent expressions in Equations (2)–(4) describe the general TNM formulation. In the present study, temperature dependence was disabled using the PolyUMod settings $\hat{\theta }=0$ and *n* = 0, while thermal expansion was suppressed by setting *α* = 0. The value $\hat{\theta }=0$ is a software convention for disabling temperature dependence and must not be substituted directly into the denominator of the general expression. For this setting, the stiffness multiplier $\left[1+\left(\theta -\theta_{0}\right)/\hat{\theta }\right]$ is taken as unity. Accordingly, the isothermal stress expressions used for the parameter settings of this study, including *q* = 0, are:

(7) $$T^{A}=\frac{\mu_{A}}{J_{A}^{e}\lambda_{A}^{e*}}\frac{L^{-1}\left(\lambda_{A}^{e*}/\lambda_{\mathrm{lock}}\right)}{L^{-1}\left(1/\lambda_{\mathrm{lock}}\right)}\mathrm{dev}\left(b_{A}^{e*}\right)+\kappa \left(J_{A}^{e}-1\right)I.$$

(8) $$T^{B}=\frac{\mu_{B}}{J_{B}^{e}\lambda_{B}^{e*}}\frac{L^{-1}\left(\lambda_{B}^{e*}/\lambda_{\mathrm{lock}}\right)}{L^{-1}\left(1/\lambda_{\mathrm{lock}}\right)}\mathrm{dev}\left(b_{B}^{e*}\right)+\kappa \left(J_{B}^{e}-1\right)I.$$

(9) $$T^{C}=\frac{\mu_{C}}{J\lambda^{*}}\frac{L^{-1}\left(\lambda^{*}/\lambda_{\mathrm{lock}}\right)}{L^{-1}\left(1/\lambda_{\mathrm{lock}}\right)}\mathrm{dev}\left(b^{*}\right)+\kappa \left(J-1\right)I.$$

Here, *μ_B_* continues to evolve according to Equation (5). With *n* = 0, the temperature multiplier in the viscoplastic flow rule in Equation (6) is also unity. Thus, temperature dependence is disabled while the strain-rate-dependent response of Networks A and B is retained. The reference temperature was fixed at *θ*_0_ = 293 K.

To determine the bulk modulus (*k*) of the material, the Young’s modulus (*E*) was estimated to be approximately 60 MPa from the tensile curve obtained at a crosshead speed of 60 mm/min. The Poisson’s ratio (ν) was assumed to be 0.49, consistent with the nearly incompressible behavior of the material [19]. Using these values, the bulk modulus was calculated as 1000 MPa according to Equation (10) and subsequently defined in the material model.

(10) $$\kappa =\frac{E}{3(1-2\nu )}$$

Equation (10) was used as a small-strain estimate to prescribe near-incompressibility in the TNM. The resulting bulk modulus of 1000 MPa was not identified from volumetric measurements and should not be interpreted as an experimentally verified volumetric property over the full deformation range. Its influence on the predicted tensile and structural responses was not quantitatively assessed and remains a limitation of the present study.

### 2.4. FEM Analysis

Nonlinear quasi-static finite element (FE) analyses of the airless tire prototypes were performed using the Static Structural module of ANSYS Workbench 2025 R1. A preliminary mesh sensitivity check was performed for the hexagonal configuration using nominal element sizes of 0.5 and 0.34 mm. Refinement increased the maximum equivalent elastic strain from approximately 1.060 to 1.095, corresponding to a 3.3% increase relative to the 0.5 mm mesh result. Considering the limited change in this monitored quantity and the substantial increase in computational time, the nominal element size of 0.5 mm was retained for all four configurations. This preliminary assessment does not establish mesh independence: load–displacement sensitivity and mesh refinement effects in the other configurations were not evaluated.

**Figure 3:**
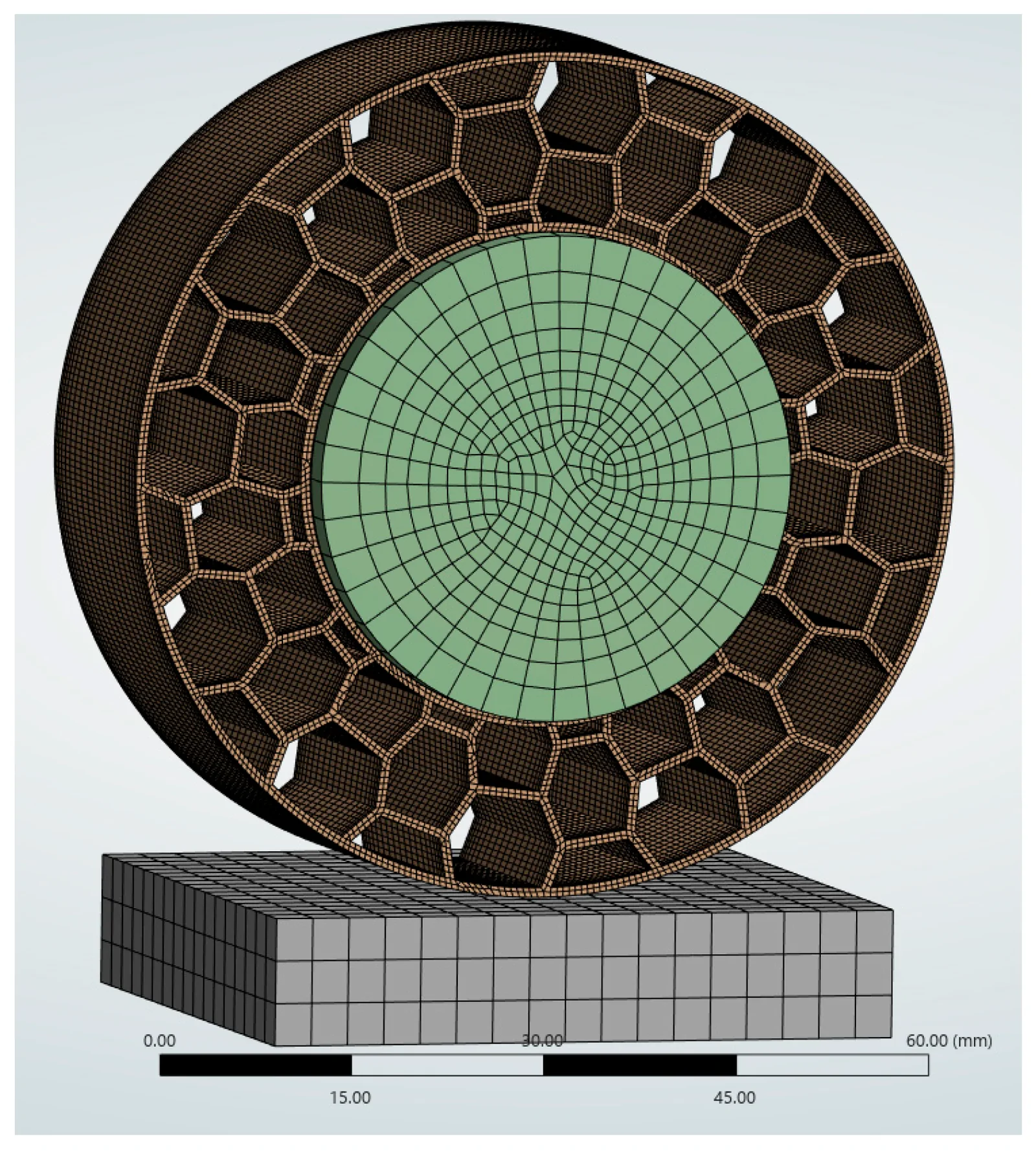
Representative finite element mesh generated for one of the four airless models considered in the numerical analyses.

The generated FE mesh is illustrated in Figure 3. Mesh quality metrics were examined to assess the suitability of the generated elements for the numerical analyses. The material parameters obtained from the calibration of experimental tensile data using ANSYS MCalibration version 25.1 were implemented in ANSYS Workbench 2025 R1 through the PolyUMod Three Network Model (TNM). To account for geometric nonlinearities associated with the large deformations of the lattice structures, the Large Deflection option was enabled.

The loading and boundary conditions were defined to reproduce the experimental compression tests. Accordingly, a prescribed vertical displacement of 10 mm was applied to the central shaft of each tire over a period of 60 s, corresponding to a constant displacement rate of 10 mm/min. The physical loading time was explicitly considered to account for the strain-rate-dependent mechanical response represented by the TNM. The bottom metallic plate was constrained using a fixed support. Frictional contact was defined between the metallic surfaces and the TPU-95A tire using a coefficient of friction of 0.50, based on the experimental findings of Vasilev et al. [24]. Furthermore, frictional self-contact was introduced to account for potential interactions between different regions of the lattice structure during deformation. A friction coefficient of 0.74 was assigned to these interfaces, consistent with the experimental results reported by Jeong et al. [25]. The numerical force–displacement responses obtained from the FE analyses were compared with the corresponding experimental compression results to evaluate the predictive capability of the numerical model.

### 2.5. Evaluation of Model Agreement

The agreement between the experimental and numerical force–displacement responses was evaluated using the normalized root mean square error (NRMSE). For each lattice configuration, the mean experimental curve obtained from three repeated compression tests was compared with the corresponding finite element prediction. The root mean square error was calculated from force values at corresponding displacement points and normalized by the difference between the maximum and minimum forces on the mean experimental curve over the evaluated displacement interval, as expressed in Equation (11).

(11) $$\mathrm{NRMSE}\left(\%\right)=\frac{\sqrt{\frac{1}{N}\sum_{i=1}^{N}(F_{\mathrm{fem},i}-F_{\exp,i}{)}^{2}}}{F_{\exp,\max}-F_{\exp,\min}}\times 100$$

*F_fem_*_,_*_i_*, *F_exp_*_,_*_i_* are the numerically predicted and experimentally measured forces at the same displacement, respectively;

*N* is the number of paired data points;

*F_exp_*_,_*_max_* *and F_exp_*_,_*_min_* denote the maximum and minimum experimental forces within the evaluated displacement interval.

## 3. Results and Discussion

**Figure 4:**
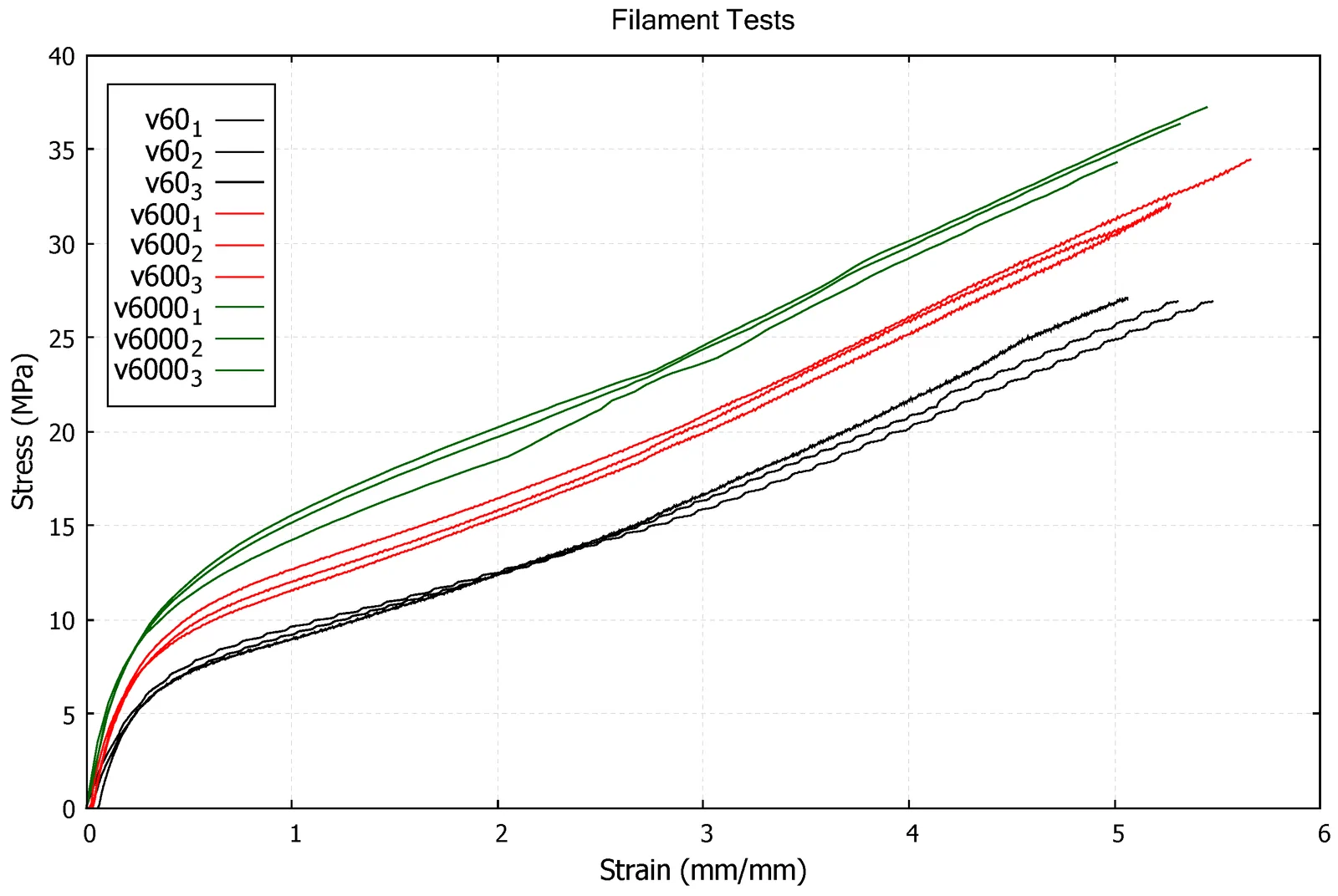
Stress–strain responses of TPU-95A filament obtained from tensile tests conducted at different loading speeds.

The mechanical behavior of TPU-95A filament was evaluated at three different loading rates by setting the crosshead speeds to 60, 600, and 6000 mm/min. Depending on the testing system’s maximum stroke, an elongation of approximately 500% was achieved in the specimens, with no failure observed. The corresponding strain rates at these crosshead speeds were calculated as 0.02, 0.2, and 2 s^−1^, respectively. Each test was repeated three times, and the resulting stress–strain curves are presented in Figure 4.

The experimental results indicate that, as the strain rate increases, the stress values of TPU-95A at the same strain level generally increase. This behavior can be attributed to the pronounced rate-dependent viscoelastic characteristics of TPU-95A, rather than considering the material solely as a time-independent elastic material. The microstructure of TPUs is characterized by a physical network comprising relatively rigid hard segments and more flexible soft segments. Since the molecular chain motions and segmental rearrangements that occur during deformation are time-dependent, an increase in strain rate reduces the time available for molecular chains to accommodate the applied deformation. Consequently, the effectiveness of molecular rearrangement and relaxation mechanisms that occur at low strain rates is reduced at higher strain rates, resulting in greater resistance to deformation. Therefore, the strain-rate dependence observed in the present study can be considered not merely as a difference between experimental curves, but rather as a material response consistent with the time-dependent molecular mobility and segmental deformation mechanisms of TPU-95A. This rate-dependent behavior is also consistent with the primary rationale for selecting the TNM used in this study. The ability of the TNM to simultaneously represent the elastic and time-dependent material responses through its different network components enables the modeling of the material response under different strain rates.

**Figure 5:**
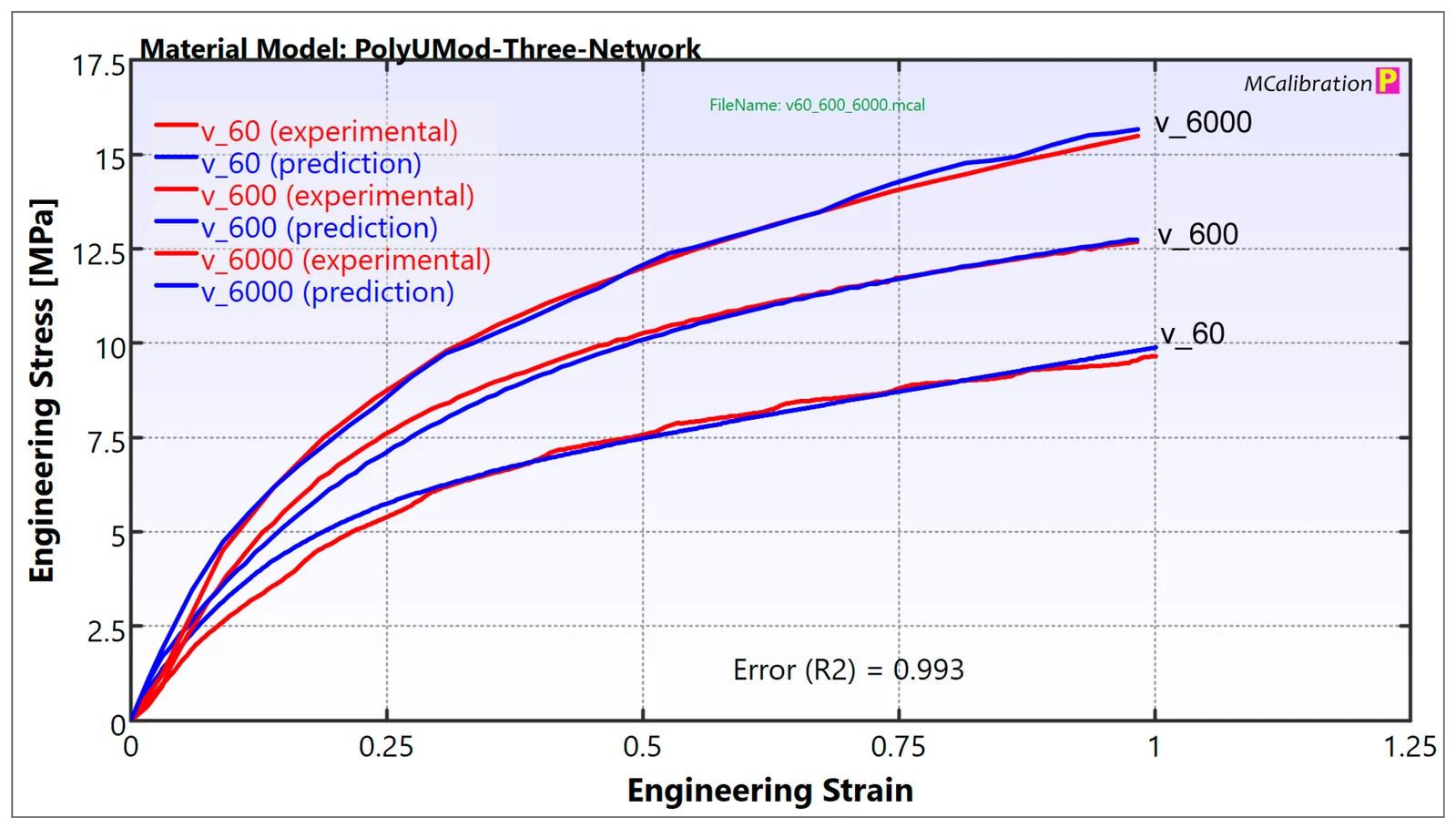
Comparison of experimental and model-predicted stress–strain curves.

The optimization process conducted in MCalibration to determine the material model parameters successfully converged after approximately 1200 iterations. The numerical data generated using the final model parameters obtained by the optimization algorithm, together with the corresponding experimental results, are presented in Figure 5 for comparison. Although the filament tensile tests were performed up to a strain of 5, preliminary finite element analyses indicated that the maximum equivalent elastic strain values of approximately 1 within the tire structure. Consequently, calibration of the Three Network Model in MCalibration was performed using only the experimental stress–strain data within the strain interval of 0–1. Restricting the calibration to this interval was intended to improve agreement between the experimental data and model predictions over the strain range relevant to the numerical analyses.

An overall coefficient of determination of 0.993 was obtained by jointly evaluating the experimental data and corresponding model predictions across all three stress–strain curves measured at different tensile speeds. This value indicates good agreement between the calibrated model responses and the experimental tensile data within the engineering strain range of 0–1. The calibrated parameters obtained at the end of the optimization process, together with the parameters that were kept fixed at their assumed values throughout the calibration process, are summarized in Table 3. The TNM parameters listed in Table 3 were identified using the Covariance Matrix Adaptation Evolution Strategy (CMA-ES) algorithm implemented in MCalibration. Parameter fitting was performed using the software’s (MCalibration version 25.1) R^2^-based fitness criterion to improve agreement between the experimental and predicted engineering stress–strain responses, simultaneously considering the tensile data obtained at loading speeds of 60, 600, and 6000 mm/min over the strain range of 0–1.

**Figure 6:**
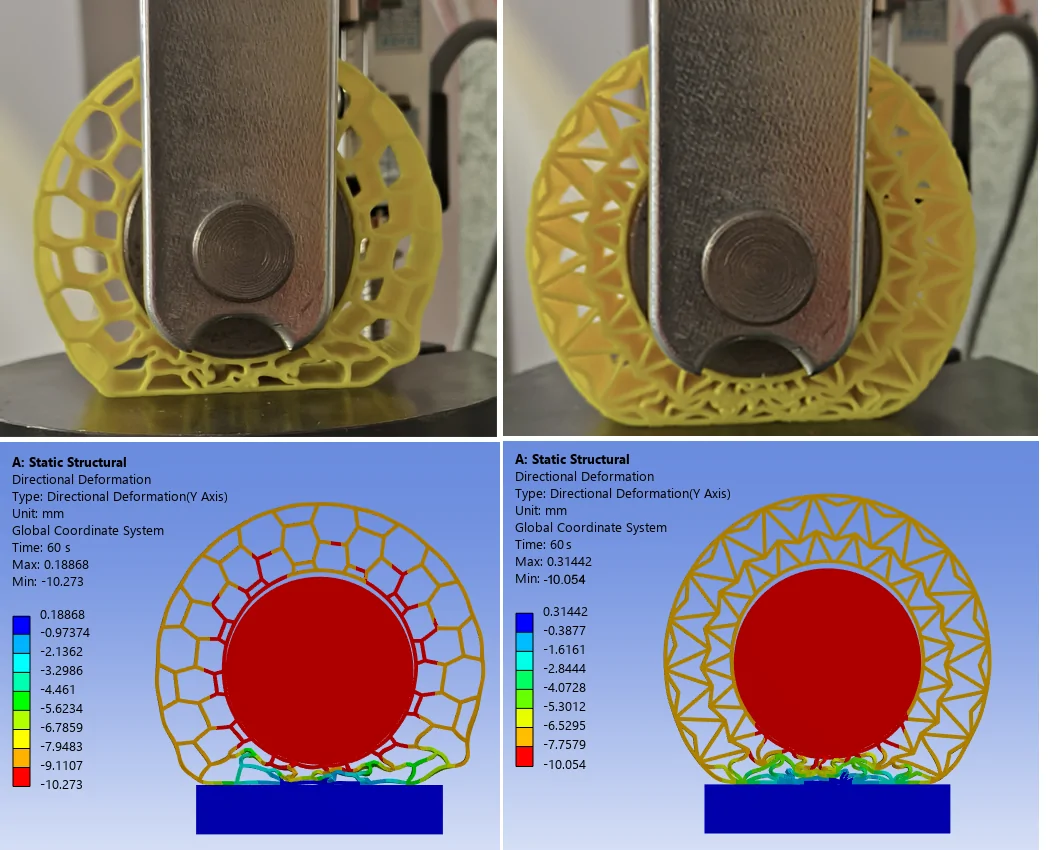
Comparison of experimentally observed and numerically predicted deformation patterns for two representative lattice-structured (hexagonal, arrowhead) airless tire designs: photographs of the deformed configurations after compression tests and corresponding deformation contours obtained from finite element analysis (FEA).

To provide a visual comparison between the experimentally observed and numerically predicted deformation behavior, representative results for two of the four lattice-structured airless tire designs are presented in Figure 6. For each selected design, the photograph showing the deformed configuration at the end of the compression test is presented together with the corresponding deformation contour obtained from the finite element analysis. These representative comparisons provide a qualitative assessment of the consistency between the experimentally observed deformation patterns and the numerical predictions.

**Figure 7:**
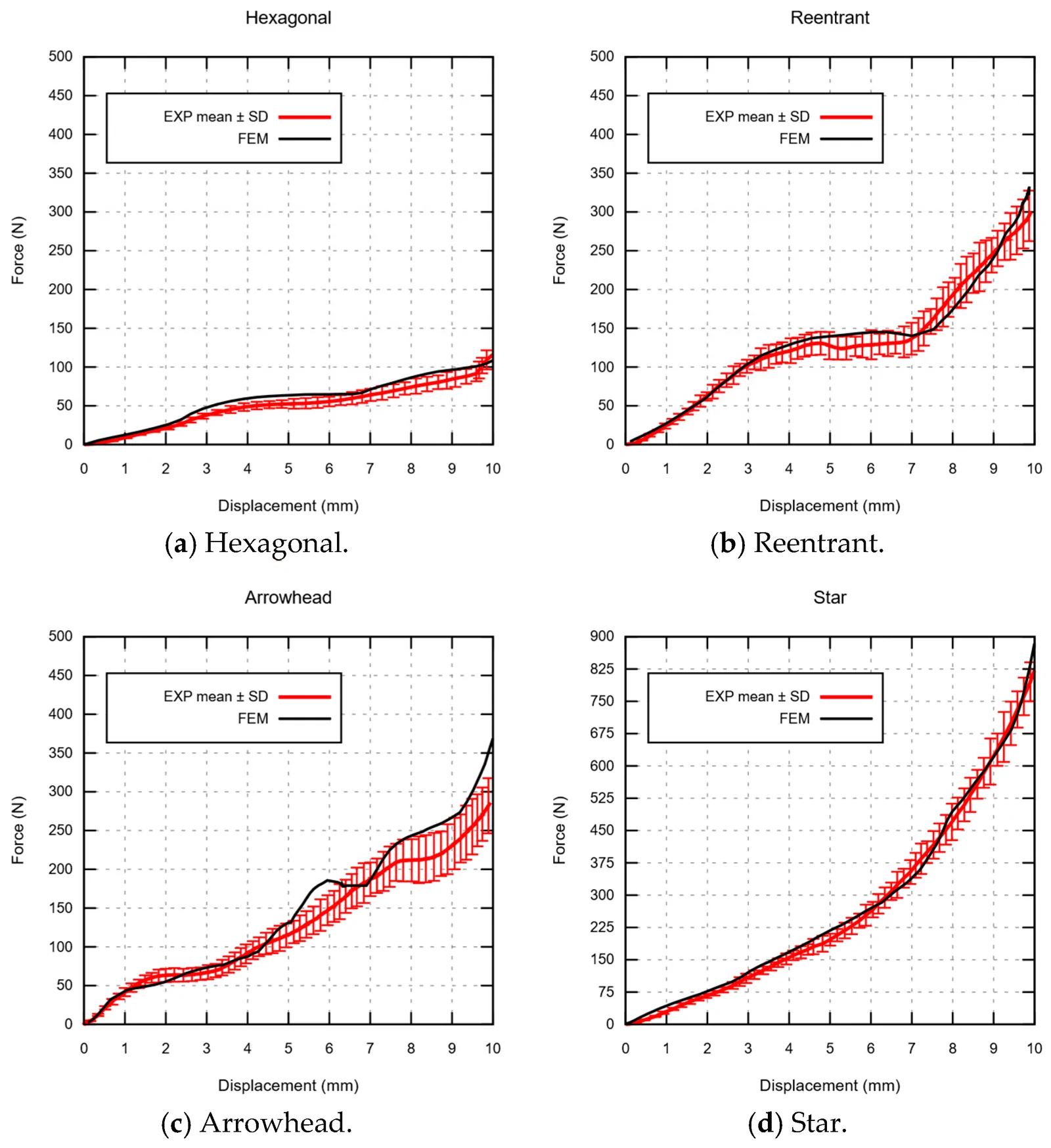
Comparison of experimental and finite element analysis (FEA) force–displacement curves for four lattice-based airless tire designs: (**a**) hexagonal, (**b**) reentrant, (**c**) arrowhead, and (**d**) star. Experimental curves represent the mean of three compression tests, with error bars indicating standard deviation.

Figure 7 compares the experimental and numerical force–displacement responses of all four lattice-structured airless tire designs, complementing the visual assessment of their deformation behavior. Each plot presents the mean experimental force–displacement curve from three repeated compression tests, along with the corresponding numerical curve from finite element analysis (FEA). This comparison enables a quantitative evaluation of the numerical model’s ability to reproduce the global mechanical response of each lattice structure under compression. The agreement between the experimental and numerical curves, particularly in the overall response trend, stiffness evolution, and force levels across the applied displacement range, provides a basis for assessing the predictive capability of the developed finite element models.

The agreement between the experimental and numerical force–displacement responses was quantitatively evaluated using the normalized root mean square error (NRMSE), as summarized in Table 4. The calculated NRMSE values were 8.23%, 11.89%, 17.61%, and 3.94% for the hexagonal, reentrant, arrowhead, and star configurations, respectively. Among the investigated geometries, the star configuration exhibited the closest agreement between the experimental and numerical responses, with the lowest NRMSE value of 3.94%. The hexagonal configuration also showed relatively good agreement, with an NRMSE of 8.23%. Higher deviations were observed for the reentrant and arrowhead configurations, with NRMSE values of 11.89% and 17.61%, respectively, indicating greater discrepancies between the experimentally measured and numerically predicted force responses over the investigated displacement range. Nevertheless, the numerical models reproduced the overall force–displacement characteristics of all four lattice configurations, although the degree of agreement depended on the lattice geometry.

The differences in NRMSE among the four configurations may reflect geometry-dependent effects of the modeling assumptions and manufacturing process. Although the same filament-calibrated TNM parameter set was used throughout, different lattice geometries generate different combinations of local tension, compression, bending, and rotation. For the arrowhead configuration, bending and rotation of the inclined members and deformation around their junctions provide plausible mechanisms through which small deviations in printed member dimensions or junction geometry could alter the global force–displacement response. Manufacturing effects are relevant because differences between experimental and numerical stiffness have been reported for FDM-printed TPU tires [8], while raster-dependent anisotropy has been demonstrated even in fully filled TPU specimens [16]. These effects are not explicitly represented by the present filament-calibrated material model and may influence each configuration differently.

Numerical discretization and contact assumptions may also contribute. Previous work on TPU95 honeycomb tire structures showed that FE modeling accuracy depends on member proportions and element formulation [12]. Consequently, a common nominal element size does not necessarily provide equivalent accuracy across all four geometries. Likewise, differences in the development of member contact and sliding may affect the influence of the adopted friction coefficients [25]. These mechanisms are possible contributors to the larger arrowhead discrepancy, rather than confirmed causes: their individual contributions were not quantified through geometric-imperfection measurements or geometry-specific sensitivity analyses. The lower NRMSE of the star configuration therefore indicates closer agreement under the investigated conditions, without establishing greater accuracy for other loading conditions.

The structural performance of the four lattice configurations was further compared using initial stiffness, force at 10 mm displacement, and loading work up to 10 mm, as summarized in Table 5. Loading work was calculated as the area under the force–displacement curve. It represents energy input during compression rather than irreversible energy dissipation, which cannot be determined without unloading data. The initial stiffness was determined from the slope of the initial approximately linear region of each force–displacement curve, using geometry-specific displacement intervals and the same procedure for the experimental and FE results.

The arrowhead configuration exhibited the highest experimental initial stiffness (41.2 N/mm), followed by the reentrant (38.5 N/mm), star (36.3 N/mm), and hexagonal (19.2 N/mm) configurations. However, the star structure provided the highest force at 10 mm (811.4 N) and the greatest loading work (2.5271 J). The reentrant structure carried a higher force at 10 mm than the arrowhead structure (321.7 versus 286.4 N), whereas its loading work was lower (0.9791 versus 1.0905 J). These differences demonstrate that initial stiffness alone does not determine performance over the full compression interval. The hexagonal configuration exhibited the lowest values for all three indicators.

The FE results reproduced the experimental ranking for loading work, whereas differences in ranking were observed for initial stiffness and force at 10 mm displacement. Nevertheless, the arrowhead configuration showed the largest relative deviations, with initial stiffness, force at 10 mm, and loading work overestimated by approximately 17.2%, 25.7%, and 28.7%, respectively. These comparisons apply to the prescribed displacement range and do not establish ultimate load capacity.

The as-received filament and FDM-printed TPU cannot be assumed to have identical microstructures or mechanical properties. The additional thermal and shear history during printing can alter molecular orientation and morphology, while interlayer bonding and microscopic voids can affect load transfer [26,27]. Furthermore, 100% infill does not guarantee isotropic behavior, as demonstrated by Martín-Sosa et al. [16]. These effects can influence constitutive parameters and their transferability to printed components [28].

The present filament-calibrated TNM treats the printed material as a homogeneous, isotropic continuum and does not explicitly account for printing-induced anisotropy or interfacial damage. The agreement with the four-tire compression tests supports this approximation under the investigated printing conditions and a loading rate of 10 mm/min. However, it does not establish material-level equivalence; broader applicability requires characterization and mechanical testing of printed specimens in different orientations.

## 4. Conclusions

This study presented an integrated experimental and numerical approach for characterizing the strain-rate-dependent mechanical behavior of TPU-95A and assessing the applicability of the calibrated model to additively manufactured lattice-based airless tires. Tensile tests were conducted at crosshead speeds of 60, 600, and 6000 mm/min using a custom-built testing system. The three averaged stress–strain curves were used simultaneously to calibrate the PolyUMod Three Network Model (TNM), representing nonlinear large-deformation behavior and strain-rate sensitivity within a single constitutive formulation. The calibration achieved an overall coefficient of determination of R^2^ = 0.993.

The calibrated parameters were subsequently used in finite element models of four airless tire configurations: a conventional hexagonal lattice and three architectures based on auxetic unit-cell geometries, namely reentrant, arrowhead, and star. Corresponding TPU-95A prototypes were manufactured by fused deposition modeling and tested under compression. The numerical models captured the overall force–displacement trends at the single compression rate tested, although agreement with the experimental results varied among the geometries. The NRMSE values were 8.23%, 11.89%, 17.61%, and 3.94% for the hexagonal, reentrant, arrowhead, and star configurations, respectively. The star configuration exhibited the closest agreement, whereas the arrowhead configuration showed the largest deviation.

Structural validation was restricted to a compression rate of 10 mm/min because the compression-testing system lacked comparable high-speed capability. Consequently, the results support the model under the investigated compression conditions, while its predictive accuracy at other compression rates remains experimentally unverified. Moreover, no direct comparison with purely hyperelastic models was performed; therefore, the present results do not establish the comparative superiority of TNM. Future studies should evaluate predictions at additional compression rates using the parameter set reported here.

A further limitation concerns the calibration dataset. Material characterization was limited to uniaxial tensile tests on 1.75 mm-diameter TPU-95A filaments, whose slender geometry poses practical difficulties for reliable compression testing and conventional biaxial testing. The uniqueness of the calibrated parameter set was not established, and no sensitivity analysis of parameters was performed. Additional compression, biaxial, and cyclic tests on suitably prepared specimens could reduce parameter uncertainty and improve assessment of the model under different stress states and loading histories. The present structural validation should therefore be interpreted in the context of the manufacturing and loading conditions investigated.

The validated FE framework provides a basis for future optimization of lattice dimensions, including cell size and strut thickness, to achieve a target stiffness while satisfying mass and manufacturing constraints. Evolutionary optimization methods, such as the strengthen elitist genetic algorithm investigated for prestressed stayed steel columns [29], could be explored for this purpose.

Computational efficiency is another consideration for future studies involving repeated simulations across lattice geometries and loading rates. Cluster-based reduced-order approaches, such as FEM-Cluster based Analysis (FCA), could be explored for this purpose. Their suitability would need to be verified against full-order FE simulations, particularly with respect to the preservation of strain-rate-dependent material behavior, large deformation, and evolving contact. Any computational savings should be assessed alongside the accuracy of the force–displacement response and relevant local deformation measures. Implementation and benchmarking of these approaches remain outside the scope of the present study.

## Figures and Tables

**Table 1:** Printing parameters used for the FDM fabrication of the airless tire prototypes.

Parameters	Value
Layer height	0.2 mm
Nozzle diameter	0.4 mm
Nozzle temperature	230 °C
Build plate temperature	60 °C
Infill density	100%
Infill pattern	Rectilinear
Printing speed	30 mm/s
Number of walls/perimeters	3
Number of top/bottom layers	4
Retraction	1 mm at 25 mm/s
Cooling fan	Off for the first three layers; 30% thereafter

**Table 2:** TNM parameters.

Symbol	Description
***µ_x_***	Shear modulus of Networks A, B, and C. While μ_A_ and μ_C_ are constant model parameters, the effective shear modulus of Network B evolves from μ_Bi_ to μ_Bf_ according to Equation (5).
${\dot{\gamma }}_{0}$	Reference flow rate; ${\dot{\gamma }}_{0}\equiv 1/s$ is adopted to ensure dimensional consistency.
*R*(*x*)	Ramp function
**𝓟_x_**	Hydrostatic pressure in the corresponding network
***a***	Pressure dependence of the flow
m_X_	Stress exponent of the corresponding network
**τ**_x_	Effective deviatoric stress of the corresponding network
${\hat{\tau }}_{x}$	Flow resistance of the corresponding network
**β**	Rate of change in the Network B modulus
${\dot{\gamma }}_{A}$	Viscoplastic flow rate in Network A
**μ_Bi_**, **μ_Bf_**	Initial and final shear modulus
$\left(\lambda_{x}^{e*}\right)$	Effective elastic chain stretch of the corresponding network
λ**_lock_**	Chain-locking stretch ratio
$J_{x}^{e}$	Elastic volume ratio of Networks A and B
***J***	Jacobian of the mechanical deformation
λ^*^	Effective chain stretch of Network C
*θ*, *θ***_0_**	Current and reference temperatures
$\hat{\theta }$	Temperature parameter controlling the temperature dependence of stiffness.
**κ**	Bulk modulus
𝓛^−1^	Inverse Langevin function
$\mathrm{dev}[b_{x}^{e*}]:$	Deviatoric part of the left Cauchy–Green deformation tensor of the corresponding network
***I***	Identity tensor
***q***	Parameter controlling the relative contribution of the I_2_ dependence in Network C
*_x_*	Index corresponding to the respective network

**Table 3:** Calibrated and fixed TNM parameters.

Calibrated Parameters	Value	Unit	Fixed Parameters	Value	Unit
*µ_A_*	8.781	MPa	$\hat{\theta }$	0	K
*λ_lock_*	8.653	-	**κ**	1000	MPa
${\hat{\tau }}_{A}$	0.842	MPa	**a**	0	-
*m_A_*	2.023	-	**n**	0	-
*µ_Bi_*	8.751	MPa	**q**	0	-
*µ_Bf_*	0.126	MPa	**α**	0	1/K
*β*	0.998	-	**θ_0_**	293	K
${\hat{\tau }}_{B}$	61.450	MPa			
*m_B_*	1.281	-			
*µc*	4.696	MPa			

Note: “**—**” denotes a dimensionless parameter. $\hat{\theta }=0$ and n = 0 disable temperature dependence in the PolyUMod TNM implementation; α = 0 suppresses thermal expansion. The corresponding isothermal stress expressions are given in Equations (7)–(9).

**Table 4:** NRMSE values between the experimental and finite element force–displacement responses of the investigated lattice-structured airless tires.

Structure	NRMSE (%)
Hexagonal	8.23
Reentrant	11.89
Arrowhead	17.61
Star	3.94

**Table 5:** Experimental and numerical comparison of initial stiffness, force at 10 mm displacement, and loading work up to 10 mm for the four lattice configurations.

Structure	Initial Stiffness (N/mm)		Force at 10 mm (N)		Loading Work to 10 mm (J)	
	EXP	FEA	EXP	FEA	EXP	FEA
Hexagonal	19.2	18.5	116.6	108.7	0.515	0.597
Reentrant	38.5	39.1	321.7	332.3	0.979	1.191
Arrowhead	41.2	48.3	286.4	360.1	1.090	1.403
Star	36.3	40.1	811.4	895.6	2.527	2.717

EXP: experimental results; FEA: finite element analysis. Loading work is the area under the force–displacement curve up to 10 mm and does not distinguish recoverable energy from irreversible dissipation.

## Data Availability

The raw data supporting the conclusions of this article will be made available by the authors on request.
